# Imperforate anus with a rectovestibular fistula and pseudotail: a case report

**DOI:** 10.1186/1752-1947-4-317

**Published:** 2010-10-07

**Authors:** Miranda D Raines, Marcia L Wills, Gretchen P Jackson

**Affiliations:** 1 Vanderbilt University School of Medicine, 2134 Fairfax Avenue Apt C-3, Nashville, TN 37212, USA; 2 Monroe Carell Jr. Children's Hospital at Vanderbilt, 2200 Children's Way, 11th Floor, Nashville, TN 37232, USA; 3 Monroe Carell Jr. Children's Hospital at Vanderbilt, 2200 Children's Way, 7100 Doctor's Office Tower, Nashville, TN 37232, USA

## Abstract

**Introduction:**

Human tails and pseudotails are rare sacrococcygeal lesions that are associated with a wide variety of anomalies and syndromes. Anorectal malformations are also relatively uncommon congenital defects that often occur in conjunction with syndromes or other congenital abnormalities. The anomalies associated with both disorders determine the timing and approach to surgical correction. We present an unusual case of a patient with both imperforate anus and a pseudotail in the absence of a syndrome or other associated anomalies and we emphasize the necessity of a thorough preoperative evaluation.

**Case presentation:**

A Caucasian girl was born at term after an uncomplicated pregnancy and was noted at birth to have a skin-covered posterior midline mass and imperforate anus with a fistula to the vaginal vestibule. Ultrasound and magnetic resonance imaging revealed a predominately fatty lesion without presacral extension and ruled out associated spinal and cord abnormalities. The patient underwent diversion with colostomy and a mucous fistula in the newborn period as a fistulogram demonstrated a long fistulous tract to normal rectum and it was anticipated that anoplasty and resection of the mass would require extensive posterior dissection. The sacrococcygeal mass was removed during posterior sagittal anorectoplasty at the age of six weeks which was determined to be a pseudotail because of the composition of brown fat and cartilage. The patient is now 14 months old with normal bowel function after a colostomy takedown.

**Conclusion:**

A comprehensive preoperative assessment and thoughtful operative plan were necessary in this unusual case because of the extensive differential diagnosis for sacrococcygeal masses in the newborn and the frequency of anomalies and syndromes associated with tail variants and imperforate anus. The pediatricians and neonatologists who initially evaluate such patients and the surgeons who correct these disorders must be aware of the potential pitfalls in their management.

## Introduction

Human tails and pseudotails are rare congenital lesions that have been reported since the late 19th century. These entities typically present as skin-covered lumbosacral masses seen in the newborn period. The possible diagnoses for this presentation are broad, ranging from benign hamartomas to aggressive malignancies and meningoceles. As one series illustrates, the management can be complicated not only by the spectrum of disorders and associations but also by parental resistance to crucial preoperative imaging [1].

Human tails may occur alone or associated with other anomalies. The most common abnormality associated with a tail is spinal dysraphism, which occurs in 49% of cases and can be diagnosed as meningocele, myelomeningocele or spina bifida. Other associated anomalies include: lipoma (27%); tethered spinal cord (20%); coccygeal vertebrae (12%); syndactyly, hemangioma; cleft palate; Crouzon syndrome; clubfoot; omphalocele; congenital tracheal stenosis; Von Recklinghausen disease; digit hypoplasia; and tetralogy of Fallot [2].

Tails and imperforate anus are both congenital anomalies that are commonly associated with syndromes. Dusmet *et al. *reported a vestigial tail in an infant with most of the major and minor defects of the VATER syndrome (see Abbreviations) including imperforate anus [3]. A true tail has also been reported in association with sirenomelia including the feature of anal atresia [4,5]. Kahler *et al. *reported a case of pygomelus (a pseudotail variant) in association with lipomyelomeningocele and an incomplete expression of Currarino's triad including anal atresia with recto-vaginal fistula [6].

We are not aware of any cases of tails or pseudotails associated with an anorectal malformation in the absence of a syndrome. We describe a newborn girl with a pseudotail and isolated imperforate anus and discuss the diagnostic evaluation necessary for operative planning.

## Case presentation

A Caucasian girl, born at 3580 g at 39 weeks and one day gestational age, was transferred to our hospital on her first day of life for evaluation and management of imperforate anus and a perineal mass. The 20-year-old mother had standard prenatal care and an uncomplicated pregnancy. She underwent ultrasonography at 21 weeks gestation and there was no evidence of congenital anomalies. The child was born by spontaneous vaginal delivery and had Apgar scores of 9 and 9 at one minute and five minutes, respectively.

**Figure 1:**
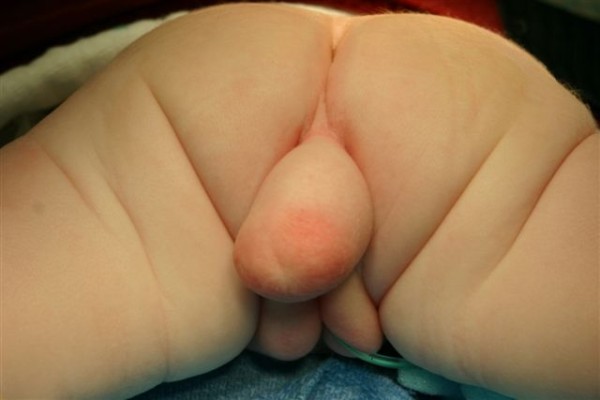
**Perineal mass**. This photograph is taken from the inferior aspect of the patient in the prone position and shows the perineal mass extending from the sacrococcygeal region prior to surgical repair.

On physical examination, she was noted to have a soft, skin-covered, midline mass, measuring 3 cm × 1 cm and emanating from the perineum posterior to the vagina (Figure 1). The child had normal external female genitalia and a sacral dimple but no anal opening. Her neurological examination was normal, as was the rest of her physical examination. She did not pass meconium on the first day of life. Ampicillin and gentamicin were given for prophylaxis. On the second day of life, she developed mild abdominal distention and flecks of meconium were noted in her diapers. Upon further inspection, a small opening was noted in the posterior aspect of the vaginal vestibule. This tract was probed with a hemostat and meconium passed through this vestibular fistula with partial decompression of the abdomen.

Anteroposterior and lateral radiographs of the sacrum and coccyx revealed normal bony structures without adjacent soft tissue calcifications. A prone, cross-table, lateral radiograph of the pelvis revealed a column of air distending the rectum and sigmoid colon, terminating just below and anterior to the caudal aspect of the sacrum.

An echocardiogram and ultrasound of the spine and retroperitoneum were done in order to assess the presence of VACTERL (see Abbreviations) anomalies known to be associated with imperforate anus. These studies were negative for cardiac, vertebral and renal anomalies. The spinal cord terminated normally at L2 and there was normal motion of cauda equina nerve roots. There was no tethered cord.

**Figure 2:**
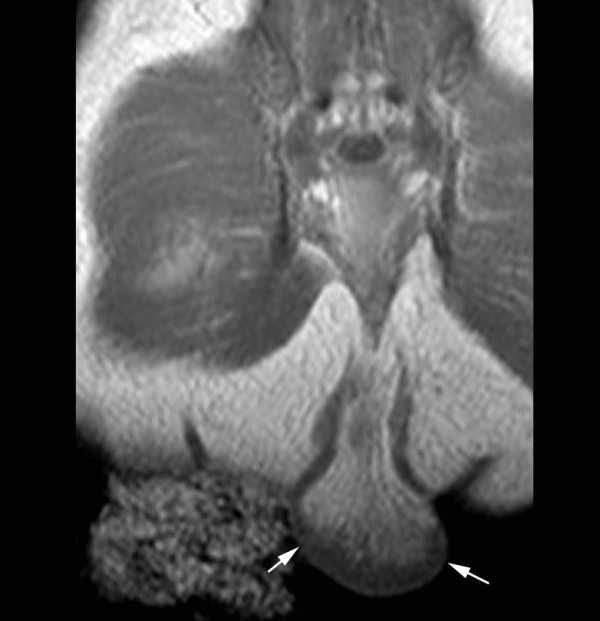
**Magnetic resonance imaging of pseudotail**. This T1 coronal image through the sacrum demonstrates the fat containing pseudotail (arrows).

**Figure 3:**
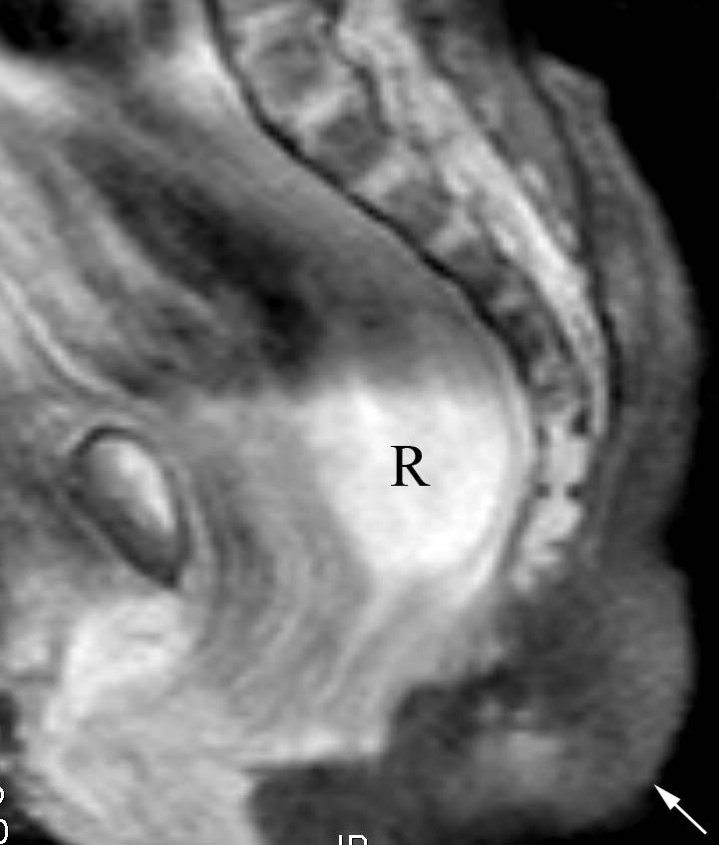
**Magnetic resonance imaging of pseudotail and sacrum**. This T2 fat-saturated coronal image demonstrates absence of a presacral mass, relatively normal appearance of the sacrum, and dilated rectum (R) in this child with known imperforate anus. The fat containing pseudotail is incompletely included (arrow).

Ultrasound was done in order to further characterize the perineal mass and it revealed a 2.5 cm × 3.6 cm × 1.3 cm lesion with internal vascular flow and a possible extension into the presacral space. These findings were suggestive of a presacral teratoma, which warranted more urgent resection. The serum alpha-fetoprotein levels were not inappropriately elevated for a newborn. Magnetic resonance imaging (MRI) revealed a pedunculated mass with predominantly fat intensity and a single central vessel (Figures 2 and 3). The structure did not have a presacral component. The vagina and rectum abutted one another and appeared to converge distally just above the perineum.

**Figure 4:**
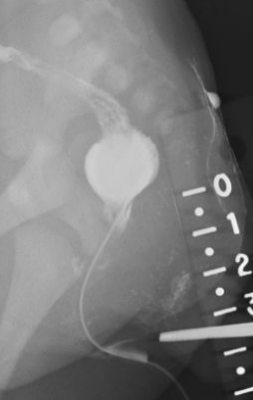
**Fistulogram**. This contrast study illustrates the long, narrow fistulous tract and distended rectum above the expected location of the anus.

Air and contrast enemas were performed through the vestibular fistula in order to evaluate the distance between the distal rectal pouch and the skin. The long, narrow, fistulous tract connected to the distended colorectal pouch, which was approximately 3 to 3.5 cm from the skin (Figure 4). The patient underwent the creation of a colostomy and mucous fistula to divert for a subsequent pull-through operation for imperforate anus. She recovered without complication and was discharged on the sixth postoperative day.

**Figure 5:**
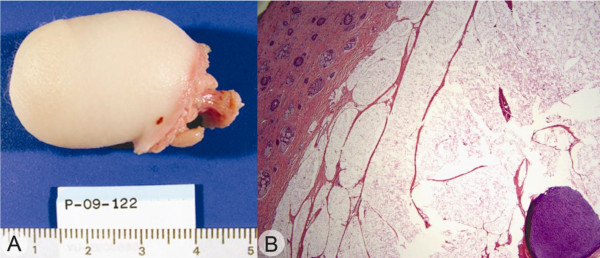
**Gross specimen**. Photograph 'A' shows hair bearing skin with underlying fat and a firm, flexible circular center area loosely attached to the fat. Representative image 'B' is the microscopic appearance of the pseudotail showing skin and adnexa with underlying brown fat and mature cartilage in the center of the circular lesion (20× total magnification).

Prior to her definitive surgical repair, a contrast enema of the distal colon through the mucous fistula was done in order to define the level of her rectum. Colostogram revealed that the distance from the rectal pouch to the perineal surface ranged from two to three vertebral body heights. There were no strictures of the colon. At six weeks of age, she underwent posterior sagittal anorectoplasty and excision of the perineal mass. A midline incision was made anterior and posterior to the perineal mass, and the skin of the mass was surrounded circumferentially. Upon palpation, the mass seemed to have a central bony structure. The subcutaneous tissues surrounding this structure were divided and the bony structure was found to extend toward the sacrum, but terminated prior to reaching it. Pathologic analysis revealed a mass composed of benign skin including normal adnexal structures with underlying brown fat and a central mature cartilage core, and it was designated a pseudotail due to its cartilage component (Figure 5). The patient recovered without complication and was discharged two days postoperatively. She underwent twice per day anal dilations until appropriate anal size for age was attained. The 14-month old toddler is now doing well with normal bowel function after colostomy takedown.

## Discussion

Dao and Netsky proposed the distinction between tails and pseudotails in 1984 [7]. A true tail is defined as the remnant of the embryonic tail, which usually regresses during the seventh and eighth weeks of gestation. A pseudotail is a protrusion from the lumbosacrococcygeal area that may be composed of normal or abnormal tissues but is not derived from the embryologic tail. Pseudotails only resemble vestigial tails in location and can represent an elongated coccyx, teratoma, lipoma, pygomelus, fibrolipoma or a prolonged sacrum [8]. True tails are covered by skin and composed of muscle, adipose, connective tissue, normal blood vessels and nerves, which are components associated with the most distal mesenchymal portion of the embryonic tail. The presence of bone, cartilage, notochord, or spinal cord tissues exclude a tail from being classified as a true or persistent vestigial tail.

This traditional classification has been recently challenged by some authors [9,10]. The designation of 'true tail' by Dao and Netsky describes the most distal portion of the embryonic tail which does not contain bone or cartilage, but the embryonic tail contains 10-12 caudal vertebrae during the fifth and sixth weeks of development [7]. Lu suggested that a tail should be classified according to whether the anomaly is associated with spinal dysraphism, since spinal dysraphism and tethered cord are the most clinically significant associations due to their propensity to cause irreversible neurologic sequelae [2].

Both human tails and imperforate anus are congenital anomalies that may occur alone or be associated with a wide range of abnormalities and syndromes. The differential diagnosis for a sacrococcygeal mass in a newborn is extensive and includes teratoma, extra-spinal ependymoma, extremely rare malignancies such as chordoma, benign entities such as lipoma and hemangioma, rectal duplication and meningocele.

Optimal operative intervention for a patient with imperforate anus and a sacrococcygeal mass requires a thorough diagnostic evaluation to define the anatomic relationships and to identify associated anomalies, such as cardiac defects, which might limit the extent of intervention that can be undertaken in the newborn period. Suspected locally aggressive tumors, such as chordomas or lesions with an age-dependent likelihood of malignancy, such as teratomas, mandate an urgent operative intervention. The correction of associated anomalies, such as spina bifida or cord tethering, can require neurosurgical expertise.

This patient had an unusual combination of disorders but she did not have significant comorbid anomalies. Diversion with subsequent anoplasty was undertaken because of the long fistulous tract which is needed to mobilize the rectum and the additional perineal dissection required to resect the likely benign tail variant.

## Conclusion

Anorectal malformations and skin-covered midline masses represent challenging diagnostic and therapeutic entities because of the extensive differential diagnoses and the array of associated anomalies. This case presents the unusual combination of imperforate anus with a rectovestibular fistula and a human pseudotail in the absence of associated anomalies or syndromes. It also illustrates the necessity for a thorough preoperative evaluation and careful therapeutic decision making.

## Abbreviations

VACTERL: an acronym representing an association of birth defects including *v*ertebral anomalies, *a*nal atresia, *c*ardiovascular anomalies, *t*racheoesophageal fistula, *e*sophageal atresia, *r*enal or *r*adial anomalies, and *l*imb defects; VATER: an acronym representing an association of birth defects including *v*ertebral anomalies, *a*nal atresia, *t*racheoesophageal fistula, *e*sophageal atresia, *r*enal or *r*adial anomalies.

## Consent

Written informed consent was obtained from the parent of the described minor patient for publication of this case report and accompanying images. A copy of the written consent is available for review by the Editor-in-Chief of this journal.

## Competing interests

The authors declare that they have no competing interests.

## Authors' contributions

MDR reviewed the literature and was primarily responsible for writing the manuscript. MLW analysed the surgical specimen, reviewed the manuscript and provided images of the specimen. GPJ oversaw the clinical care of this patient, performed the operations and made revisions to the manuscript. All authors have read and approved the final manuscript.
